# Supplementary material for: A Critical View of the Application of the APEX Software (Aqueous Photochemistry of Environmentally-Occurring Xenobiotics) to Predict Photoreaction Kinetics in Surface Freshwaters
Source: Molecules. 2019 Dec 18;25(1):9. doi: 10.3390/molecules25010009 (PMC7017383; doi:10.3390/molecules25010009)
Supplement: Supplementary file 1 [file molecules-25-00009-s001.zip › 3.2.4_gcc-4.4.0.zip!/3.2.4_gcc-4.4.0/3.2.4_gcc-4.4.0/doc/octave/html/liboctave/Concept-Index.html]

Concept Index - Octave C++ Classes


Next: Function Index,
Previous: Bugs,
Up: Top


---

## Concept Index

- acknowledgements: Acknowledgements
- arrays: Arrays
- bounds: Bounds
- bugs, known: Bugs
- collocation weights: Collocation Weights
- contributors: Contributors
- copyright: Contributors
- DAE: Differential Algebraic Equations
- factorizations: Matrix Factorizations
- installation: Installation
- installation trouble: Bugs
- integration: Quadrature
- introduction: Introduction
- known causes of trouble: Bugs
- linear Constraints: Linear Constraints
- matrix factorizations: Matrix Factorizations
- matrix manipulations: Matrix and Vector Operations
- NLP: Nonlinear Programming
- nonlinear Constraints: Nonlinear Constraints
- nonlinear equations: Nonlinear Equations
- nonlinear functions: Nonlinear Functions
- nonlinear programming: Nonlinear Programming
- numerical integration: Quadrature
- objective functions: Objective Functions
- ODE: Ordinary Differential Equations
- optimization: Optimization
- orthogonal collocation: Collocation Weights
- QP: Quadratic Programming
- quadratic programming: Quadratic Programming
- quadrature: Quadrature
- ranges: Ranges
- troubleshooting: Bugs
- vector manipulations: Matrix and Vector Operations
- warranty: Contributors
